# The gut microbiota-brain connection: insights into major depressive disorder and bipolar disorder

**DOI:** 10.3389/fpsyt.2024.1421490

**Published:** 2024-11-05

**Authors:** Jia Zhao, Jiaoyan Liu, Jianguo Feng, Xing Liu, Qinxue Hu

**Affiliations:** ^1^ Department of Critical Care Medicine, The Affiliated Hospital, Southwest Medical University, Luzhou, China; ^2^ Department of Anesthesiology, The Affiliated Hospital, Southwest Medical University, Luzhou, China; ^3^ Anesthesiology and Critical Care Medicine Key Laboratory of Luzhou, The Affiliated Hospital, Southwest Medical University, Luzhou, China

**Keywords:** major depressive disorder (MDD), bipolar disorder (BD), gut microbiota (GM), therapeutic options, microbe-gut-brain axis (MGBA)

## Abstract

Major depressive disorder (MDD) and bipolar disorder (BD) are two of the most prevalent mood disorders that seriously jeopardize both physical and mental health. The current diagnosis of MDD and BD relies primarily on clinical symptoms. However, correctly differentiating between MDD and BD during depressive episode states remains a substantial clinical challenge. The human gut hosts a large and diverse microbiota, which plays a pivotal role in various physiological processes. Emerging evidence suggests that the gut microbiota (GM) exerts beneficial effects on mental health disorders, including MDD, BD, and schizophrenia, through the microbe-gut-brain axis (MGBA). In recent years, the relationship between GM and mood disorders has garnered considerable attention, leading to intensive research in this area. The MGBA is a bidirectional communication system between the gut and the brain. Growing evidence indicates that the brain can influence the GM, which in turn may modulate the brain through this axis. This review aims to explore the changes in the GM of patients with MDD and BD and evaluate the effects of different treatments on their GM, including medication, probiotic, prebiotic and synbiotic interventions, and fecal microbiota transplantation (FMT). By doing so, we seek to identify potential disease-specific biomarkers, improve differential diagnosis, and offer novel therapeutic avenues for these disorders.

## 1 Introduction

Major depressive disorder (MDD) and bipolar disorder (BD) are two important subtypes of severe mood disorders that pose a significant global disease burden and economic stress. The 2010 Global Burden of Disease report on mental disorders revealed that MDD contributes 40.5% to the global burden of mental disorders, while BD accounts for 7.0% (1). MDD is characterized by distinct episodes lasting at least 2 weeks, involving disruptions across various domains of emotional, cognitive, and neurotrophic functioning (2). The lifetime prevalence of MDD is estimated at 10.8% (3). BD is characterized by recurrent episodes of elevated mood and depression accompanied by fluctuations in activity and energy, along with distinct cognitive, physical, and behavioral symptoms (4). The lifetime risk of BD is approximately 1% (5). Current treatment options for mood disorders include pharmacological interventions, such as antidepressants, and non-pharmacological interventions, including psychotherapy (6). However, each approach has several limitations, for example, drug therapy often proves ineffective and is associated with a high risk of relapse (7). Therefore, there is an urgent need to develop new therapeutic strategies to enhance treatment outcomes.

The gut microbiota (GM) is a dynamic and complex microbial ecosystem that includes bacteria, viruses, archaea, protozoa, and fungi (8). It communicates with the host and plays an important role in maintaining human health (9). The microbe-gut-brain axis (MGBA) represents the bidirectional communication pathway between the gut and the brain, primarily mediated through the immune system, the vagus nerve, and the endocrine system (8, 10). The gut microbiota influences brain function by modulating neurotransmitters and their precursors, as well as by secreting and upregulating key proteins and metabolites involved in the release of neuropeptides and gastrointestinal hormones, including short-chain fatty acids (SCFAs) and brain-derived neurotrophic factor (BDNF) (11).

Over the past decade, MGBA has garnered increasing attention and has been implicated in various disorders, including MDD and BD (12). Evidence suggests that patients with mood disorders exhibit alterations in GM composition (9, 13, 14), and these changes correlate with the severity of mood disorders (15).Conversely, psychobiotics, prebiotics, and fecal microbiota transplantation (FMT) have shown potential in the treatment of mood disorders by modulating the GM and enhancing MGBA function (16). In patients with MDD, dysregulation of the GM can disrupt bile acid metabolism, potentially triggering depressive symptoms through systemic inflammation (17). Thus, MGBA dysfunction may play an important role in the pathogenesis of mood disorders. Recently, there has been a growing interest in GM regulation as a therapeutic strategy for mood disorders. However, these treatments remain in the developmental stage, and pharmacological interventions remain the primary therapeutic approach. In addition, the etiological overlap and symptomatic similarities between MDD and BD complicate differential diagnosis, thereby delaying treatment and negatively affecting patient prognosis (18, 19).Despite emerging insights, little is known about the shared and distinct microbiological characteristics of MDD and BD. Therefore, this review aims to describe the specific GM alterations in patients with MDD and BD, providing insights into potential biomarkers for diagnosis and treatment. Moreover, the interactions between various treatment modalities and GM are explored to elucidate underlying mechanisms.

## 2 GM diversity in MDD and BD

Alpha and beta diversity are widely utilized in sequencing-based microbiota studies to offer comprehensive insights into the structure and composition of microbial communities (20). Alpha diversity measures the diversity within a single sample’s GM and is commonly used to assess both the richness (abundance) and evenness (distribution) of unique taxa. Low alpha diversity is frequently associated with potential health risks for the host organism (20, 21). Beta diversity is calculated from pairwise measures of similarity or dissimilarity between GM in different groups and is used to evaluate compositional differences between samples, such as between patients and healthy controls (HCs) (20, 21). We then compared gut microbial diversity between patients with MDD and BD using both alpha and beta diversity metrics.

Findings on alpha and beta diversity in patients with MDD have been mixed. Overall, several studies have reported no significant differences in alpha diversity between people with MDD and HCs (2, 21–27). However, some studies have reported decreased alpha diversity in patients with MDD (28–30). In addition, using the Shannon index, Jiang et al. and Ye et al. observed increased alpha diversity in patients with MDD (31, 32). Unlike alpha diversity, most studies have shown that beta diversity in patients with MDD differs significantly from that of HCs (2, 21, 24, 28, 33). However, similar to alpha diversity, some studies have reported conflicting results regarding beta diversity in individuals with MDD. In a study of 160 adolescent participants, no significant differences in beta diversity were observed among MDD patients, suggesting that age may influence beta diversity in MDD (34).

In patients with BD, alpha diversity is reduced compared to healthy individuals, and the extent of this reduction is positively associated with the duration of the disease (35). This reduction may be attributed to the inflammatory processes associated with BD, leading to progressive neurobiological and functional deterioration, or to the chronic use of psychotropic medications and potential malnutrition (36, 37). In contrast to MDD, no significant differences in beta diversity were observed in patients with BD compared to HCs (38).

In summary, compared to HCs, patients with MDD exhibited increased beta diversity, while those with BD showed decreased alpha diversity. A comparative study of MDD and BD yielded results consistent with those of studies comparing both groups to HCs (38, 39). These findings suggest a potential avenue for distinguishing MDD from BD during episodes of depressive symptoms. However, given the inconsistency of results across studies, this conclusion remains a preliminary diagnostic indicator. More reliable biomarkers are needed for a more accurate and precise diagnosis.

## 3 GM abundance in MDD and BD

### 3.1 Changes in GM abundance in MDD

Most studies have shown that the GM of patients with MDD differs from that of healthy individuals at the phylum, class, order, family, and genus levels, with particular emphasis on differences at the phylum, family, and genus levels (**Table 1**).

**Table 1:** Changes of GM abundance in patients with MDD.

Author and year	Sample	Age (years, mean ± SD)	Gender (female%)	Phylum	Family	Genus
Xiao et al. 2024 (40)	MDD (N=44) HC (N=42)	MDD: 32.39 ± 13.26 HC: 33.21 ± 13.05	MDD: 68.18 HC: 66.67	NA	*Enterobacteriaceae ↑* *Prevotellaceae ↓*	*Agathobacter, Clostridium ↓*
Dong et al. 2022 (41)	MDD (N = 63) HC (N = 30)	MDD: 28.34 ± 8.63 HC: 29.23 ± 6.59	MDD: 68.3 HC: 66.7	*Actinobacteria ↑*	*Bifidobacteriaceae ↑ Lactobacillaceae ↓*	*Agathobacter, Bifidobacterium, Blautia ↑*
Caso et al. 2021 (52)	a-MDD (N = 46) r-MDD ((N = 22) HC (N = 45)	a-MDD: 42.10 r-MDD: 45.85 HC: 44.72	a-MDD: 78.26 r-MDD: 77.27 HC: 75.50	NA	NA	*Alistipes*, *Bilophila* ↑ *Anaerostipes*, *Dialister* ↓
Lai et al. 2021 (33)	MDD (N = 26) HC (N = 29)	MDD: 43.73 ± 11.46 HC: 39.41 ± 10.96	MDD: 69.23 HC: 55.17	*Actinobacteria ↑ Bacteroidetes ↓*	NA	*Bifidobacterium ↑*
Zhang et al. 2021 (42)	MDD (N =36) HC (N = 45)	MDD: 36.81 ± 13.52 HC: 39.29 ± 11.44	MDD: 41.67 HC: 57.78	NA	*Actinomycineae, Bacteroidaceae, Porphyromonadaceae, Rikenellaceae ↑ Prevotellaceae ↓*	*Bacteroides*, *Parabacteroides*, *Alistipes* ↑ *Prevotella*, *Eggerthella* ↓
Liu et al. 2020 (25)	MDD (N =43) HC (N = 47)	MDD: 21.9 ± 2.1 HC: 22.1 ± 1.8	MDD: 88.40 HC: 72.30	*Bacteroidetes ↑ Firmicutes ↓*	*Ruminococcaceae ↓*	*Flavonifractor ↑* *Ruminococcus*, *Faecalibacterium ↓*
Zheng et al. 2020 (39)	Discovery set: MDD (N = 122) BD (N =169) HC (N = 171) Validation set: MDD (N = 43) BD (N = 48) HC (N = 46)	Discovery set: MDD: 26.54 ± 4.07 BD: 25.59 ± 8.41 HC: 26.85 ± 5.48 Validation set: MDD: 37.13 ± 9.15 BD: 21.87 ± 7.44 HC: 45.4 ± 7.1	Discovery set: MDD: 63.11 BD: 49.70 HC: 58.48 Validation set: MDD: 67.44 BD: 35.42 HC: 47.83	*Bacteroidetes ↑ Proteobacteria -*	*Bacteroidaceae, Bifidobacteriaceae, Lachnospiraceae ↑ Enterobacteriaceae ↓*	*Bacteroides ↑* *Roseburia, Ruminococcus ↓*
Chen et al. 2021 (43)	MDD (N =62) HC (N = 46)	MDD: 39.58 ± 12.66 HC: 36.93 ± 8.58	MDD:100 HC:100	*Bacteroidetes, Proteobaeteria, Fusobacteria ↑ Firmicutes , Actinobacteria ↓*	*Rikenellaceae, Porphyromonadaceae, Oscillospiraceae, Corynebacteriaceae ↑ Ruminococcaceae, Lachnospiraceae*, *Eubacteriaceae, Lactobacillaceae* ↓	*Eggerthella*, *Streptococcus ↑* *Oscillibacter*, *Bacteroides ↓*
Yang et al. 2020 (44)	Discovery set: MDD (N =118) HC (N =118) Validation set: MDD (N=38) HC (N=37)	Discovery set: MDD: 27.19 ± 4.71 HC: 26.86 ± 5.24 Validation set: MDD: 37.07 ± 9.45 HC: 36.39 ± 10.75	Discovery set: MDD: 56.78 HC: 56.78 Validation set: MDD: 86.84 HC: 64.86	NA	NA	*Bacteroides* ↑ *Blautia , Eubacterium ↓*
Chung et al. 2019 (24)	MDD (N =36) HC (N =37)	MDD: 45.83 ± 14.08 HC: 41.19 ± 12.73	MDD: 82.35 HC: 62.16	*Actinobacteria, Firmicutes ↑ Bacteroidetes ↓*	*Bifidobacteriaceae, Lachnospiraceae ↑ Prevotellaceae ↓*	*Bifidobacterium, Blautia*, *Eggerthella, Parabacteroides, Ruminococcus, Streptococcus ↑ Prevotella ↓*
Huang et al. 2018 (45)	MDD (N =27) HC (N =27)	MDD: 48.7 ± 12.8 HC: 42.3 ± 14.1	MDD: 74.07 HC: 74.07	*Firmicutes ↓*	*Lachnospiraceae, Ruminococcaceae ↓*	*Prevotella, Faecalibacterium ↓*
Lin et al. 2017 (46)	MDD (N =10) HC (N =10)	MDD: 36.2 ± 10.1 HC: 38.1 ± 2.9	MDD: 40.00 HC: 40.00	*Firmicutes ↑ Bacteroidetes ↓*	NA	*Prevotella, Klebsiella, Streptococcus*, *Clostridium XI ↑*
Jiang et al. 2015 (31)	a-MDD (N =29) r-MDD (N =17) HC (N =30)	a-MDD: 25.3 ± 5.4 r-MDD: 27.1 ± 5.4 HC: 26.8 ± 5.4	a-MDD: 38.00 r-MDD: 47.00 HC: 50.00	*Actinobacteria, Bacteroidetes, Proteobacteria↑* *Firmicutes ↓*	*Enterobacteriaceae ↑ Lachnospiraceae, Ruminococcaceae ↓*	*Blautia*, *Phascolarctobacterium, Clostridium XIX ↑* *Prevotella*, *Ruminococcus ↓*
Naseribafrouei et al. 2014 (27)	MDD (N=37) HC (N=18)	MDD: 42.9 ± 13.9 HC: 46.1 ± 13.9	MDD: 54.05 HC: 61.11	*Bacteroidetes ↓*	*Lachnospiraceae ↓*	*Alistipes, Oscillibactergenus ↑*

MDD, major depressive disorder; BD, bipolar disorder; HC, Healthy Control; a, active disorder group; r, response group; ↑, upregulated; ↓, downregulated; -, no change; NA, not available.

In the 14 included studies, we found that the changes at the phylum level in patients with MDD primarily involved *Actinobacteria, Bacteroidetes, and Firmicutes*. Although some studies reported opposite findings, the overall trend indicated an increase in *Actinobacteria* and *Bacteroidetes* levels, along with a decrease in *Firmicutes* levels (25, 31, 41). These variations may be influenced by the age of patients with MDD. Compared to age-matched HCs, younger patients showed elevated *Bacteroidetes* and reduced *Firmicutes*, while middle-aged patients exhibited the opposite pattern. Notably, *Actinobacteria* levels consistently increased, independent of age (47).

At the family level, patients with MDD exhibited decreased levels of *Lachnospiraceae* and *Ruminococcaceae*, along with increased levels of *Bifidobacteriaceae* (24, 27, 31, 39, 41). It has been hypothesized that since most of the patients in the study were in the early stages of MDD, the elevation of *Bifidobacteria* may be a protective response strategy of the GM, which enhances intestinal barrier function, anti-inflammatory, and immunomodulatory effects, as well as promotes gamma-aminobutyric acid (GABA) production (41, 48).

At the genus level, we observed trends consistent with those at the phylum and family levels: a consistent decrease in the levels of *Firmicutes*-*Ruminococcaceae*–*Ruminococcus* and *Firmicutes*-*Lachnospiraceae*-*Blautia*, along with a consistent increase in *Actinobacteria-Bifidobacteriaceae-Bifidobacterium* (25, 28, 43). Notably, *Actinobacteria-Bifidobacteriaceae-Bifidobacterium* demonstrated highly concordant elevated levels across studies, which is rare among other genera. Furthermore, a study examining the GM of subgroups with varying severities of MDD revealed distinct GM phenotypes in patients with moderate and severe MDD. The phylum *Actinobacteria* emerged as a covariant marker, with the genera *Collinsella, Eggerthella, Alistipe, Faecaliba*, and *Framonifractor suggested as* potential diagnostic indicators for MDD (15). However, it is important to note that *Bifidobacterium* counts can increase with higher plant-based protein intake (49). The elevation of the phylum *Bacteroidetes* is primarily attributed to the combined effects of increased levels of *Bacteroidetes-Bacteroidaceae-Bacteroides* and decreased levels of *Bacteroidetes-Prevotellaceae-Prevotella (* 25, 31, 46). Elevated levels of *Bacteroides* are associated with cytokine production, aligning with findings of upregulation of pro-inflammatory bacteria and downregulation of anti-inflammatory bacteria in MDD patients, thus providing support for the inflammatory hypothesis of depression (44, 50). Reduced levels of *Prevotella* are associated with the development of autism in young patients (46). *Klebsiella*, a gram-negative bacterium, may play a significant role in the pathophysiology of MDD through its translocation and the immune responses to lipopolysaccharide (LPS) (51). In the study by Lin et al., the changes in the relative proportions of *Prevotella* and *Klebsiella* in the fecal flora were expected to be a valuable indicator for laboratory diagnosis and prognostic assessment of patients with MDD (46). Another study showed that individuals with MDD had elevated levels of *Bilophila* and *Alistipes* compared to HCs. The LPS present on the membranes of *Bilophila* and *Alistipes* can induce depressive symptoms through the activation of toll-like receptor 4. Additionally, *Alistipes* may influence the production of indole, which affects tryptophan metabolism and disrupts the homeostasis of the 5-hydroxytryptaminergic system (52). Indole derivatives are known neuroinhibitory molecules, and indoles along with their derivatives participate in “MGBA” mediated metabolic, immune, and neural communication processes by binding to the aryl hydrocarbon receptor, contributing to the onset of MDD (53). However, due to the limited literature included in this study, it is not yet possible to draw definitive conclusions about the specific changes in *Alistipes*. Studies have shown that an increase in *Blautia* abundance is accompanied by an increase in serum C-reactiveprotein levels. Therefore, *Blautia* may cause MDD by promoting inflammation (54).

### 3.2 Changes in GM abundance in BD

Studies have shown that the relative abundance of the *Actinobacteria* phylum and its associated families is increased in patients with BD. Both *Actinobacteria* and *Coriolobacteriaceae* are involved in lipid metabolism and correlate with cholesterol levels, which may contribute to obesity in patients with BD (36). Research has found that the microbiota of patients with BD and higher Body Mass Index (BMI) harbored a significantly greater quantity of *Lactobacilli* than the group with lower BMI. Additionally, the family *Lactobacillaceae* and the genus *Lactobacillus* were more abundant in BD patients with metabolic syndrome, suggesting that *Lactobacilli* may also play a role in obesity among these patients (8). In addition, BMI is positively correlated with *Roseburia* abundance in patients with BD (14)*. Ruminococcaceae* have been reported to be associated with energy metabolism pathways, including gluconeogenesis, glycolysis, and pentose phosphate pathways. Therefore, lower levels of *Ruminococcaceae* in patients with BD may be linked to abnormal glucose metabolism (14).

Untreated patients with BD have been found to exhibit downregulated levels of various butyrate-producing bacteria compared to HCs, including *Roseburia*, *Faecalibacterium*, and *Coprococcus*. These bacteria are responsible for producing SCFAs, such as butyrate, which can influence CNS function. In particular, butyrate has been shown to affect hippocampal function and promote the expression of BDNF, a protein associated with antidepressant-like effects in animal models. Therefore, a deficiency in butyrate-producing bacteria may be linked to the development of BD (14).

*Faecalibacterium* is a prevalent intestinal gram-positive microorganism known for its anti-inflammatory properties (55). In patients with BD, a decrease in *Faecalibacterium *levels appears to be associated with disease severity, psychotic symptoms, and altered sleep quality. Furthermore, *Faecalibacterium* shows a correlation with self-reported symptoms and disease severity (56); therefore, it may be possible to differentiate patients with BD from HCs based on *Faecalibacterium* levels (47). *Enterobacter* spp. have also been found to positively correlate with serum interleukin-6 (IL-6) levels, and BD is strongly associated with immune dysfunction. Consequently, *Enterobacter* spp. may contribute to the pathogenesis of BD through mechanisms related to immune dysfunction (57) (**Table 2**).

**Table 2:** Changes of GM abundance in patients with BD.

Author and year	Sample	Age (years, mean ± SD)	Gender (female%)	BMI (kg/m^2^)	Phylum	Family	Genus
Huang et al. 2023 (57)	BD (N=72) HC (N=16)	BD: 24.16 ± 9.26 HC: 42.75 ± 11.22	BD:45.83 HC:56.25	BD: 21.39 HC: 21.89	NA	NA	*Bacilli*, *Lactobacillales*, *Veillonella ↑* *Dorea ↓*
Lai et al. 2021 (33)	BPD (N = 25) HC (N = 28)	BPD: 36.92 ± 10.14 HC: 39.21 ± 11.11	BPD:44.00 HC:53.57	BPD: 22.11 HC: 21.14	*Actinobacteria, Firmicutes ↑* *Bacteroidetes ↓*	NA	*Firmicutes, Actinobacteria ↑ Bacteroidetes ↓*
Zheng et al. 2020 (39)	Discovery set: MDD (N = 122) BD (N =169) HC (N = 171) Validation set: MDD (N = 43) BD (N = 48) HC (N = 46)	Discovery set: MDD: 26.54 ± 4.07 BD: 25.59 ± 8.41 HC: 26.85 ± 5.48 Validation set: MDD: 37.13 ± 9.15 BD: 21.87 ± 7.44 HC: 45.4 ± 7.1	Discovery set: MDD:63.11 BD:49.70 HC:58.48 Validation set: MDD:67.44 BD:35.42 HC:47.83	Discovery set: MDD: 22.41 BD: 21.77 HC: 22.07 Validation set: MDD: 22.08 BD: 25.06 HC: 24.07	*Proteobacteria ↑* *Bacteroidetes ↓*	*Pseudomonadaceae ↑*	NA
Hu et al. 2019 (14)	BD (N = 52) HC (N = 45)	BD: 24.15 ± 9.50 HC: 36.29 ± 12.22	BD:48.08 HC:48.89	BD: 21.58 HC: 22.37	*Bacteroidetes ↑* *Firmicutes ↓*	*Ruminococcaceae ↓*	*Roseburia, Faecalibacterium, Coprococcus ↓*
Painold et al. 2019 (36)	BD (N = 32) HC (N = 10)	BD: 41.31 ± 14.73 HC: 31.40 ± 7.61	BD:43.75 HC:60.00	BD: 28.44 HC: 24.26	*Actinobacteria ↑*	*Coriobacteriaceae ↑* *Ruminococcaceae ↓*	*Faecalibacterium ↓*
Lu et al. 2019 (58)	BD (N = 36) HC (N = 27)	BD: 32.64 ± 10.643 HC: 28.89 ± 11.095	BD:41.67 HC:44.44	BD: 22.16 HC: 21.84	NA	NA	*Faecalibacterium prausnitzii, Bacteroides-Prevotella group, Atopobium Cluster, Enterobacter spp, Clostridium Cluster IV ↑*
Coello et al. 2019 (59)	BD (N = 113) HR (N=39) HC (N = 77)	BD: 31 HR: 28 HC: 29	BD:62.5 HR:53.8 HC:61.0	BD: 24.80 HR: 24.40 HC: 24.20	NA	NA	*Flavonifractor ↑*
Evans et al. 2017 (56)	BD (N = 115) HC (N = 64)	BD: 50.2 ± 12.8 HC: 48.6 ± 16.6	BD:72.2 HC:62.5	BD: 29.30 HC: 26.00	NA	NA	*Faecalibacterium ↓*

MDD, major depressive disorder; BD, bipolar disorder; HC, Healthy Control; BPD, bipolar disorder with current major depressive episode; BMI, Body Mass Index; HR, their healthy first degree relatives; ↑, upregulated; ↓, downregulated; -, no change; NA, not available.

### 3.3 Identical changes in GM abundance between MDD and BD

*Enterobacteriaceae* levels have been found to be elevated in both MDD and BD (60), with an inflammatory state in the GM facilitating the proliferation of *Enterobacteriaceae* (61). Some studies have reported that the family *Lactobacillaceae* and members of the genus *Lactobacillus* are enriched in MDD and BD. In the authors’ exploratory analysis, *Lactobacillaceae* levels were significantly increased only in the medication group, suggesting that the use of psychotropic drugs may contribute to the elevation of this family and its member genus in patients (38).

At the genus level, both MDD and BD exhibited relatively increased levels of *Flavonifractor*, a genus of bacteria involved in the breakdown of quercetin, a flavonoid known for its antioxidant and anti-inflammatory properties, which has been shown to have depression-relieving effects (8, 62). However, *Flavonifractor* may also induce oxidative stress and inflammation in its host (59), suggesting that increased levels could contribute to the inflammation associated with depression. Additionally, *Clostridium* levels were found to be elevated in both conditions. Adults with depression and those on specific antidepressants are at a higher risk of developing *Clostridium difficile* infections (63).

Depletion of anti-inflammatory butyrate-producing bacteria and enrichment of pro-inflammatory bacteria, lower levels of SCFA-producing bacterial genera, higher levels of lactate-producing bacteria, and higher levels of bacteria associated with glutamate and GABA metabolism were found in both diseases (21, 60). SCFAs exert anti-inflammatory effects by interfering with the NF-κB pathway, and a reduction in SCFA-producing bacteria may cause MDD and BD via an inflammatory response (54). They may be considered biomarkers to improve diagnostic accuracy, guide treatment, and help monitor the response to therapy. However, further studies are needed to determine its feasibility.

### 3.4 Differential changes in GM abundance between MDD and BD

Interestingly, some taxa are differentiated between MDD and BD. *Bacteroidaceae*, *Veillonellaceae*, and *Roseburia* are higher in MDD than in BD, while *Enterobacteriaceae*, *Pseudomonadaceae*, and *Megasphaera* are higher in BD than in MDD (21, 39). In a study exploring GM differences among MDD, bipolar disorder with the current major depressive episode (BPD) and HCs, the genera *Escherichia* and *Klebsiella* showed changes in abundance only between the BPD and HC groups. At the species level, compared with BPD patients, MDD patients had a higher abundance of *Prevotellaceae* including *Prevotella denticola F0289*, *Prevotella intermedia 17*, *Prevotella ruminicola*, and *Prevotella intermedia*. Furthermore, the abundance of *Fusobacteriaceae*, *Escherichia blattae DSM 4481* and *Klebsiella oxytoca* were significantly increased, whereas the *Bifidobacterium longum subsp. infantis ATCC 15697 = JCM 1222* was significantly reduced in the BPD group compared with MDD group (64). MDD is usually characterized by higher *Alistipes* and *Parabacteroides* and lower *Prevotella*; BD is usually characterized by higher *Bifidobacterium* and *Oscillibacter* (21).

## 4 Changes in GM after treatment

### 4.1 Pharmacotherapy and GM

Pharmacological interventions remain the cornerstone of depression treatment. Antidepressants are broadly categorized into selective serotonin reuptake inhibitors (SSRIs), serotonin-norepinephrine reuptake inhibitors (SNRIs), tricyclic and tetracyclic antidepressants, atypical antidepressants, monoamine oxidase inhibitors, N-methyl-D-aspartate (NMDA) antagonists, and neuroactive steroids, such as GABA-A receptor positive modulators. Among these, second-generation antidepressants, particularly SNRIs, are the most frequently prescribed (65, 66). These antidepressants not only alleviate depression through their respective mechanisms of action but also significantly impact the GM (50, 67, 68). Antidepressants reduce the gut bacterial abundance and increase beta diversity, with significant reductions in the abundance of *Ruminococcus*, *Adlercreutzia*, and the unclassified genus *Alphaproteobacteria* in particular (69). In patients with MDD treated with escitalopram, alpha diversity of the gut microbiota decreased to levels comparable to healthy controls. In addition, the abundance of *Christensenellaceae_R-7_group*, *[Eubacterium]_ruminantium_group*, and *Fusobacterium* was significantly elevated, while *Lactobacillus* abundance, as well as the *Firmicutes/Bacteroidetes* ratio, were significantly reduced compared to pre-treatment levels and healthy controls. Interestingly, higher pre-treatment levels of *Firmicutes* were positively associated with treatment response (70, 71). Duloxetine, an SNRI, exerts its antidepressant effects primarily through modulation of gene expression in the cortex. Specifically, it inhibits the upregulation of mitochondrial oxidative phosphorylation genes and downregulates genes related to neuronal plasticity. This action is closely associated with the downregulation of *Ruminococcus* abundance (69). In addition, the relative abundance of *Blautia*, *Bifidobacterium*, and *Coprococcus* has been positively correlated with the antidepressant efficacy of SSRIs (72).

Current pharmacological treatments for BD include lithium, atypical antipsychotics (AAP), and antiepileptic drugs. Lithium, considered the first-line treatment for BD, has been shown to increase the GM species richness and diversity, with significant increases in the relative abundance of *Clostridium* spp., *Clostridium perfringens*, *Enterobacter* spp., and *Christenellaceae* spp. Atypical antipsychotics, such as aripiprazole, quetiapine, and olanzapine, are widely used in the management of acute manic and depressive episodes, as well as for the maintenance therapy in BD. In a study involving aripiprazole administration in rats, an increase in the relative abundance of *Clostridium* spp., *Clostridium tumefaciens*, *Enterobacter* spp., and *Eubacterium faecalis* was observed in the gut following treatment (8). In addition, another study demonstrated that olanzapine reduced the *Mycobacterium avium*/*Hypobacterium chauvinum* ratio, contributing to increased appetite, visceral fat accumulation, and peripheral inflammation, all of which are risk factors for obesity (8). Similarly, reduced gut biodiversity in BD patients following quetiapine treatment, especially the diminished relative abundance of *Ackermannia* and *Suturella* associated with normal metabolism—has been linked to APP-induced obesity and metabolic complications (8, 55, 73). Antiepileptic drugs, including valproic acid, lamotrigine, and carbamazepine, are frequently used to stabilize mood in BD. In one trial, valproic acid was associated with increased abundance of *Clostridium* spp., *Clostridium perfringens*, *Enterobacter* spp., and *Christenellaceae* spp. in the rat cecum (74). Additionally, a vitro study found that lamotrigine significantly inhibited the growth of Gram-positive bacteria, such as *Bacillus subtilis* and *Staphylococcus aureus* (55). However, the relationship between microbiological alterations induced by antiepileptic drugs and therapeutic efficacy in BD remains to be further elucidated.

Ketamine, an NMDA receptor antagonist, has shown potent efficacy in the treatment of BD and in antidepressant-naïve patients with MDD (75). It has been shown that ketamine modulates the dysbiotic composition of the GM by elevating the abundance of *Actinobacteriaceae* and *Coriolobacteriaceae Piliobacteriaceae*, *Lactobacillus*, *Turicibacter*, and *Sarcina*, while decreasing levels of *Fusobacterium*, *Clostridium* and *Ruminalococcaceae*, *Clostridium* and *Butyric acidomonas* spp. These changes in the GM are associated with improvements in depressive symptoms (75–78).

Collectively, these studies suggest that pharmacological treatments can significantly alter the abundance and composition of the GM. Moreover, the composition and abundance of gut microbes appear to affect the efficacy of pharmacological treatments and are closely related to certain adverse effects, such as metabolic complications (79).

### 4.2 Non-pharmacotherapy and GM

Although pharmacotherapy is effective in treating MDD and BD, it’s often accompanied by withdrawal symptoms, gastrointestinal adverse reactions, and even life-threatening side effects (80). In addition, between 1/3 to 1/2 of MDD patients do not respond to multiple antidepressants (81). In contrast, non-pharmacological interventions with significantly fewer side effects, such as electroconvulsive therapy, psychosocial interventions, cognitive-behavioral therapy, and dietary-dietary fiber or probiotic therapies, are receiving increasing attention and research.

#### 4.2.1 Probiotic, prebiotic and synbiotic interventions

**Figure 1:**
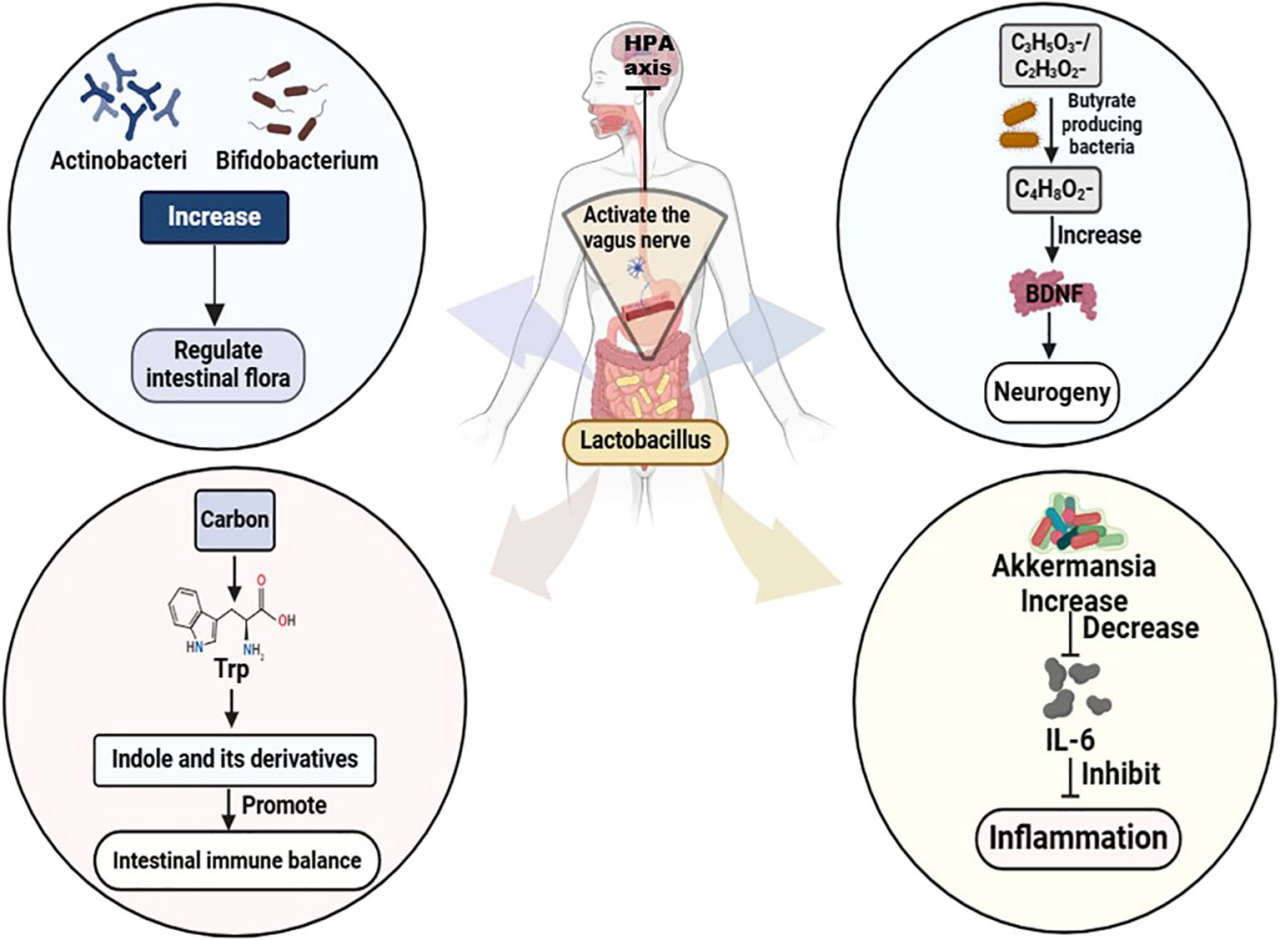
Mechanism of Lactobacillus treating MDD and BD. Trp, Tryptophan; HPA, hypothalamic-pituitary-adrenal; C3H5O3-/C2H3O2-, Lactate/acetate; C4H8O2-, Butyrate; BDNF, brain-derived neurotrophic factor; IL-6, Interleukin-6.

Probiotics, prebiotics, synbiotics, and postbiotics exert a potent regulatory effect on the GM (82). Studies have demonstrated that administration of probiotic powder containing *Lacticaseibacillus paracasei strain Shirota* (LcS) to patients with MDD or BD improves depressive symptoms. This improvement is positively correlated with an increased abundance of *Actinobacteriophage* and *Bifidobacterium* in the GM (83). Probiotic supplementation with strains such as *Lactobacillus* and *Bifidobacterium bifidum* has been shown to elevate serotonin levels and reduce both depression and rumination scores in patients with BD, as well as decrease rehospitalization rates, duration of hospital, and mania scores in BD patients with manic episodes (84). Moreover, *Lactobacilli* or *bifidobacteria* produce significant amounts of lactate and/or acetate, which are subsequently metabolized to butyrate by butyrate-producing bacteria such as *Clostridium butyricum* and *E.faecalis przewalskii*. This process enhances brain-derived neurotrophic factor (BDNF) levels, promotes neurogenesis, and ultimately alleviates depressive symptoms in MDD (83). In addition, Lactobacillus can convert sugar-derived carbon sources into tryptophan, producing indole and its derivatives, which promote hippocampal neurogenesis and reduce depressive symptoms (85). Probiotic intake has also been shown to downregulate systemic pro-inflammatory cytokines such as interleukin-1beta, tumor necrosis factor-alpha, IL-6, and interferon-gamma (86). An 8-week intervention with *Lactobacillus plantarum PS128* in MDD patients found that improvements in depressive symptoms were accompanied by an increase in the relative abundance of *Akkermansia*, a bacterium closely related to IL-6 and suppressed inflammation (87, 88). *Akkermansia* may further alleviate depression by modulating the GM composition and metabolites, which in turn upregulate molecules associated with pathological changes in depression (corticosterone, dopamine, and BDNF) as well as antidepressant markers (β-alanyl-3-methyl-L-histidine and edaravone) (89). In addition, *Lactobacillus testosteroni La 1* or *LcS* activates gastric vagal afferents, leading to inhibition of the hypothalamic-pituitary-adrenal (HPA) axis and subsequent reduction of renal sympathetic nerve activity. Meanwhile, *Lactobacillus short left SBC 8803* promotes small intestinal 5-hydroxytryptamine secretion, thereby activating the intestinal branch of vagal afferents. Collectively, these findings suggest that probiotic strains modulate the composition and metabolites of the intestinal microflora, promoting neurogenesis, inhibiting inflammatory responses, and modulating the neuroendocrine system to downregulate the stress-induced activation of the HPA axis, thereby controlling depressive symptoms (83) (**Figure 1**).

Elevated activity in the tryptophan metabolic pathway within the circulating microbiome of non-responders to antidepressant therapy has been associated with reduced availability of tryptophan, the sole precursor for serotonin synthesis. This reduction consequently impairs serotonin production (71). A prospective open trial suggested that *Clostridium butyricum MIYAIRI 588* in combination with antidepressants is effective in improving depressive symptoms in patients with antidepressant-resistant MDD (90). Based on the role of *Lactobacillus* in increasing tryptophan levels and promoting neurogenesis, we hypothesize that a combination of *Lactobacillus* and antidepressants may offer more pronounced improvements in depressive symptoms for patients with antidepressant-resistant MDD than the combination with *Clostridium butyricum*. In another clinical trial, a probiotic/magnesium spirulina formula (comprising *Lactobacillus acidophilus*, *Bifidobacterium bifidum*, *Streptococcus thermophilus*) used as an adjunct to SSRI intervention in patients with drug-resistant MDD led to significant improvements in depressive symptoms and quality of life, whereas after discontinuing the probiotic adjuvant, patients’ depressive symptoms recurred (91). These findings suggest that, in addition to ameliorating depressive symptoms via multiple pathways, probiotics may serve as a potent adjunctive agent in improving antidepressant drug resistance and inhibiting the recurrence of depression.

#### 4.2.2 FMT

FMT is considered a generally safe treatment with minimal adverse effects, which is achieved by transferring feces from a healthy donor to a patient with GM disorders to directly restore the GM composition of the recipient (92, 93). FMT has demonstrated efficacy in the treatment of recurrent *Clostridium difficile* infections and has also shown therapeutic potential in several diseases associated with intestinal microbiota dysregulation, such as ulcerative colitis, irritable bowel syndrome, and hepatic encephalopathy (94). Given the crucial role of the MGBA in regulating mood, behavior, and cognition, and the involvement of GM disturbances in the pathogenesis of depression, FMT holds promise as a potential therapy for depression (95). Moreover, FMT in patients with MDD or BD can induce depressive-like symptoms. It has been shown that FMT from MDD patients or rodents with depressive-like behaviors can induce similar behaviors in recipient rodents through systemic inflammation. Notably, severing the vagus nerve prevents this effect, as well as the antidepressant effects of SSRIs (96). Another study showed that activation of NACHT, LRR and PYD structural domain protein 3 inflammatory vesicles led to a decrease in *Trichoderma*, *Ruminalococcaceae*, and *Prevotella*, while promoting an enrichment of *Mycobacterium anisopliae*, resulting in depression-like symptoms (97). These results suggest that the MGBA may exert bidirectional effects and participate in the onset and progression of depression. In a clinical study, oral administration of FMT capsules to depression patients with irritable bowel syndrome resulted in a significant increase in bacterial alpha diversity and the abundance of bacterial communities, predominantly *Bacteroides immitis* and *Bacteroides thicketi*, alongside significant improvements in depressive symptoms (95). In addition, spinal cord injured depressed rats showed a significant reduction in depressive and anxiety-like behaviors following FMT from healthy rats (97). These results indicate a potential positive role for FMT in the treatment of depression; however, there remains a notable lack of research on its effects in patients with MDD or BD.

## 5 Conclusion and perspective

MDD and BD are significant contributors to the global disease burden, often resulting in severe cognitive impairment that substantially affects patients’ social functioning and quality of life (97). Gut microbes can influence brain function through neural, immune, and metabolic pathways, either directly via the vagus nerve or indirectly via gut- and microbial-derived metabolites, as well as gut hormones and endocrine peptides, and disruptions in their composition are strongly associated with the development of depressive behaviors (98). This review explores the altered composition of gut microorganisms in patients with MDD and BD, aiming to identify specific microbial signatures that could serve as therapeutic targets for probiotic and FMT (33, 99). Furthermore, the positive effects of pharmacological treatment, probiotic interventions, and FMT in restoring the composition of the gut microflora and thus improving depressive symptoms, as well as possible mechanisms, are elucidated.

Participants with MDD exhibit altered beta diversity, while those with BD show reduced alpha diversity, which may serve as a distinguishing feature between the two. The specific alterations in gut flora as potential biomarkers need to be further investigated. Additionally, the role of gut microbiota-derived metabolites represents a promising avenue for future research, offering new insights into pathogenic mechanisms and potential therapeutic strategies.

Antidepressant non-responders demonstrate a reduced abundance of *Firmicutes* and elevated levels of tryptophan metabolism (71). Meanwhile, butyrate-producing bacteria have shown a positive effect in the treatment of antidepressants non-responders, possibly due to their role in promoting of neurogenesis (33). The multifaceted effects of *Lactobacillus* in enhancing neurogenesis and increasing tryptophan levels suggest that it may be a more effective adjunctive treatment for individuals who do not respond to antidepressants. However, this hypothesis requires further investigation to be validated. Improving the abnormal composition of the microflora and modulating the microflora network to down-regulate tryptophan metabolism is a possible direction to fundamentally improve antidepressant resistance. Although conventional probiotic treatments, primarily composed of *Bifidobacterium* spp. and *Lactobacillus* spp., are generally considered safe, their efficacy in fully correcting microbiota dysbiosis remains suboptimal (99). FMT offers a more direct approach to restoring the disturbed microbial composition, and the similarity between the recipient’s and donor’s microbiota after transplantation is positively correlated with therapeutic outcomes. However, the evidence supporting FMT in patients with MDD and BD remains limited (95). Given the vast complexity of the human microbiota network and the current lack of comprehensive studies, further research is urgently needed to advance our understanding of microbiota regulation as a therapeutic strategy.
